# Quality of Life and Return to Work After Radiotherapy in Young Adults With Head‐and‐Neck Cancer—A Bicentric Cross‐Sectional Observational Study

**DOI:** 10.1002/cam4.71502

**Published:** 2025-12-29

**Authors:** Charlotte Pannenbecker, Clemens Seidel, Jiadai Zou, Daniel Schnell, Gunnar Wichmann, Christoph Becker, Sabine Klagges, Henning Schäfer, Andreas Knopf, Andreas Dietz, Anca‐Ligia Grosu, Anja Mehnert‐Theuerkauf, Nils H. Nicolay, Alexander Rühle

**Affiliations:** ^1^ Department of Radiation Oncology University Medical Center Leipzig Leipzig Germany; ^2^ Comprehensive Cancer Center Central (CCCG) Germany, Partner Site Leipzig Leipzig Germany; ^3^ Department of Radiation Oncology, Medical Center – University of Freiburg, Faculty of Medicine University of Freiburg Freiburg Germany; ^4^ Clinic for Otorhinolaryngology and Head and Neck Surgery, Department of Head Medicine and Oral Health University of Leipzig Leipzig Germany; ^5^ Department of Otorhinolaryngology, Head and Neck Surgery, Medical Center – University of Freiburg, Faculty of Medicine University of Freiburg Germany; ^6^ Clinical Cancer Registry Leipzig Region (CCRL) Leipzig Germany; ^7^ Department of Medical Psychology and Medical Sociology University Medical Center Leipzig Leipzig Germany

**Keywords:** AYA, chemotherapy, head‐and‐neck cancer, patient‐reported outcomes, psycho‐oncology, quality‐of‐life, radiotherapy, return‐to‐work

## Abstract

**Objective:**

Young adults (18–45 years) with head‐and‐neck cancer represent a unique population with limited data on quality of life (QoL) and return‐to‐work after radiotherapy. This bicentric study aimed to evaluate these outcomes.

**Methods:**

Conducted at two comprehensive cancer centers, the study included young head‐and‐neck cancer survivors treated with curative radiotherapy between 2003 and 2023. QoL was assessed with EORTC QLQ‐C30 and HN43; distress, depression, and anxiety with the NCCN Distress Thermometer, PHQ‐9, and GAD‐7; fear of cancer progression and work ability with FoP‐Q‐SF and WAI.

**Results:**

Out of 83 eligible patients, 58 (70%) participated. The median age at radiotherapy was 41 years, with a balanced gender distribution (40% female, 60% male). The median time from radiotherapy to questionnaire completion was 82.5 months. Mean global QoL was 65.0, comparable to the age‐ and gender‐matched reference population (67.2). Clinically relevant distress was reported by 52%, severe depressive symptoms by 12%, moderate‐to‐severe anxiety by 21%, and strong fear of cancer progression by 38%. At the time of the study, 66% had returned to work. Those who returned to work reported lower symptom scores, and less depression, anxiety, and distress. In the multiple regression analysis, gender was significantly associated with return to work, with females showing higher odds of returning.

**Conclusions:**

While overall QoL was comparable to the general population, young head‐and‐neck cancer survivors face psychological and work reintegration challenges. Returning to work is associated with improved QoL and reduced psychological symptoms, highlighting the need for tailored survivorship care.

## Graphical abstract

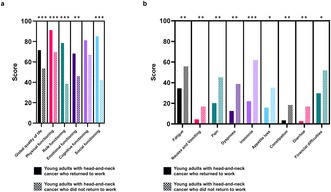

## 1 Introduction

Head‐and‐neck cancer is newly diagnosed in more than 900,000 people and results in about 500,000 deaths per year worldwide [1]. Surgery and radiotherapy are the main treatment modalities for localized head‐and‐neck cancer. Head‐and‐neck cancer is uncommon in young adults, representing < 10% of all head‐and‐neck cancer patients in most developed countries [2, 3]. However, recent epidemiological analyses revealed an increasing proportion of young adults with oral cavity cancer [2, 3, 4]. Young adults diagnosed with head‐and‐neck cancer represent a special population, characterized by distinctive clinical and psychosocial factors. For instance, the proportion of oral cavity cancer patients, especially those with tongue cancer, without a history of smoking or alcohol use is higher in this population, and there appears to be a rising incidence among young women in recent years [5]. Anxiety, depression, and financial distress are reported to be more common in young adults with head‐and‐neck cancer compared with their older counterparts [6, 7]. Their age places them in a transitional phase of life, where they are often navigating significant developmental milestones, educational pursuits, career aspirations, and interpersonal relationships [8]. Consequently, the impact of cancer on their lives extends more significantly beyond predominantly medical concerns [9]. Previous studies that analyzed the psychological situation of adolescents and young adult cancer survivors after acute treatment did report long‐term challenges in work, education, and finances [8, 10].

Radiotherapy, either alone or with concurrent systemic treatment, can result in long‐lasting chronic toxicities in surviving head‐and‐neck cancer patients which negatively affect their quality of life and also their ability to return to work [11, 12]. Emotional distress and fear of cancer progression have been reported to be prevalent in the majority of patients with head‐and‐neck cancer [13]. However, very little is known to what extent these patient‐reported outcomes are present in young adults with head‐and‐neck cancer. Understanding the unique characteristics and needs of young adults with head‐and‐neck cancer is essential for delivering tailored, comprehensive care and optimizing their long‐term well‐being. To the best of our knowledge, there is yet no study available that comprehensively assessed quality of life, distress, fear of cancer progression, and return to work in young adults with head‐and‐neck cancer. Therefore, we aimed to analyze these patient‐reported outcome measures within a bicentric cross‐sectional observational study.

## 2 Subjects and Methods

### 2.1 Study Design

This cross‐sectional observational study was performed at the Department of Radiation Oncology, University Medical Center Leipzig, Germany, and the Department of Radiation Oncology, University Medical Center Freiburg, Germany. Inclusion criteria for this study were (i) at least one course of radiotherapy, either alone or with concurrent systemic treatment, with curative intention for a head‐and‐neck cancer between 2003 and 2023, (ii) age 18–45 years at the time of first radiotherapy, (iii) being alive at January 1, 2024, (iv) the ability to understand the German questionnaires, and (v) informed consent to participate in this study. Although young adults are traditionally defined as those under 40 years old, we selected an age range of 18–45 years based on existing literature about young adults with head‐and‐neck cancer, which often includes patients up to 45 years of age [5, 9, 14, 15]. The study was approved in advance by the local ethics committees (reference numbers 077/23‐ek [Leipzig], and 371/20 with the amendment number 240159 [Freiburg]) and was performed in accordance with the declaration of Helsinki. This study followed the Strengthening the Reporting of Observational Studies in Epidemiology (STROBE) guidelines for reporting observational studies (see Appendix S2).

Between January and April 2024 (Leipzig) and in August 2024 (Freiburg), patients were contacted via telephone and asked for participation in the study. If patients agreed, they received the informed consent form and questionnaires via post. All patients that were included in our analysis provided written informed consent. Patient and treatment characteristics of both participants and nonresponders were collected retrospectively from electronic medical records.

### 2.2 Questionnaires

Paper‐based questionnaires were used in our study. Quality of life was measured with the European Organization for Research and Treatment of Cancer (EORTC) QLQ‐C30 [16] questionnaire and the organ‐specific QLQ‐HN43 module [17]. Our objective was to compare the results of the five functioning scales (physical, role, cognitive, emotional, and social), three symptom scales (fatigue, pain, and nausea/vomiting), global quality of life, and six single‐item measures (dyspnea, insomnia, appetite loss, constipation, diarrhea, and financial difficulties) between our patients and the age‐ and gender‐adjusted general German population. For this, we utilized updated reference data from the EORTC QoL Group [18].

Distress was assessed with the German version of the National Comprehensive Cancer Network (NCCN) Distress Thermometer ranging from 0 (no distress) to 10 (extreme distress) in the past week [19]. A score of ≥ 5 was considered a clinically significant distress level. Depression and anxiety were measured using the German versions of the Patient Health Questionnaire‐9 (PHQ‐9) [20] and Generalized Anxiety Disorder Screener (GAD‐7) [21], respectively. The PHQ‐9 yields a total score ranging from 0 to 27, with thresholds of ≥ 5, ≥ 10, and ≥ 15 indicating mild, moderate, and severe depression, respectively. GAD‐7 scores range from 0 to 21, categorizing anxiety levels as follows: 0–4 indicates minimal anxiety, 5–9 mild anxiety, 10–14 moderate anxiety, and 15–21 severe anxiety. Fear of cancer progression was measured with the 12‐item short version of the Fear of Progression (FoP‐Q‐SF) questionnaire [22].

In our study, we used the German translation of the Work Ability Index (WAI) to assess self‐reported work ability [23]. The questionnaire evaluates various aspects, including work demands, health status, and resources. It comprises seven items that measure current work ability, work ability in relation to the physical and mental demands of the job, the number of physician‐diagnosed diseases, the estimated impairment due to health status, sick leave over the past 12 months, a self‐prognosis of work ability, and mental resources [24]. The WAI score ranges from 7 to 49 points, calculated by summing the seven items, with higher scores indicating better work ability. Scores are categorized into four classes: poor (7–27 points), moderate (28–36 points), good (37–43 points), and excellent (44–49 points).

To the best of our knowledge, no validated German questionnaire on return to work is currently available. To address this gap, we developed a tailored questionnaire, incorporating selected items from internationally recognized return to work questionnaires, which were slightly adapted for our purposes (see Appendix S1). The goal was to assess changes in patients' work life following cancer treatment and to identify potential barriers preventing their return to work.

### 2.3 Statistical Analysis

Statistical analyses were conducted using IBM SPSS Statistics version 29 (IBM Corp., Armonk, NY, USA), while results were visualized using GraphPad Prism software version 10.2.3 (GraphPad Software, San Diego, CA, USA). Descriptive statistics were utilized to present the study cohort. Mann–Whitney *U* tests were performed to compare quality of life outcomes between two groups, while unpaired two‐sided *t*‐tests were used to compare the quality of life of our cohort with the age‐ and gender‐matched German population [18]. To explore the multivariable relationships between return to work and covariates, a linear regression with return to work as the dependent variable was performed. Here, listwise deletion of missing data was performed. Given the exploratory nature of our analyses, adjustments for multiple testing were not undertaken. A significance level of *p* < 0.05 was adopted for all analyses.

## 3 Results

### 3.1 Characteristics of the Study Cohort

A total of 83 patients met the inclusion criteria and were deemed potentially eligible for the study. Among them, 58 provided consent to participate and were subsequently included in the analysis (Figure S1). Detailed patient and treatment characteristics of participants as well as of patients who declined participation or did not send back the questionnaires are shown in Table 1 and Table S1, respectively. The median age of participants at the start of their first radiotherapy session was 43 years (IQR, 35–44), with a balanced gender distribution (23 females and 35 males). The most common tumor sites were the oral cavity (*n* = 21; 36%), oropharynx (*n* = 12; 21%), and salivary glands (*n* = 7; 12%). Thirteen patients (22%) underwent definitive radiotherapy, while 45 patients (78%) received radiotherapy following primary surgery. Among these 45 patients, 21 (47%) received postoperative radiotherapy alone, while 24 (53%) underwent postoperative chemoradiotherapy. All patients successfully completed their prescribed radiotherapy courses, with a median of 32.5 fractions (IQR, 30–33). The median time from the last radiotherapy fraction to questionnaire completion was 82.5 months (IQR, 48.0–134.5).

**TABLE 1:** Patient characteristics and return to work information of study participants (*n* = 58).

	Median (IQR)
Age at start of first radiotherapy course [years]	41 (35–44)
Radiotherapy treatment fractions	32.5 (30–33.25)
Median time between last fraction of radiotherapy and study participation [months]	82.5 (48–134.5)

		*n*	%
Gender	Male	35	60.3
	Female	23	39.7
Education	Secondary school	32	55.2
	High school diploma	8	13.8
	University degree	10	17.2
	Others	8	13.8
Health insurance	Public health insurance	54	93.1
	Private health insurance	4	6.9
Partnership at the time of radiotherapy	Partner	39	67.2
	No partner	19	32.8
Partnership at the time of study participation	Partner	35	60.3
	No partner	21	36.2
	Unknown	2	3.4
Smoking status at the time of radiotherapy	Never smoker	20	34.5
	Former smoker	27	46.6
	Smoking during radiotherpay	9	15.5
	Unknown	2	3.4
Performance status at the start of radiotherapy	ECOG 0	35	60.3
	ECOG 1	22	37.9
	Unknown	1	1.7
Primary cancer site	Oral cavity	21	36.2
	Oropharynx	12	20.7
	Hypopharynx	2	3.4
	Larynx	3	5.2
	Nasopharynx	5	8.6
	Salivary gland	7	12.1
	Nasal cavity/paranasal sinus	4	6.9
	Head‐and‐neck cancer of unknown primary	3	5.2
	Multilevel pharynx	1	1.7
Type of radiotherapy	Definitive	13	22.4
	Adjuvant	45	77.6
Concurrent systemic treatment	Concurrent systemic treatment	37	63.8
	No concurrent systemic treatment	21	36.2
Radiotherapy‐induced chronic grade 3/4 toxicity	No	34	58.6
	Yes	11	19.0
	Unknown	13	22.4
Cancer recurrence	No	52	89.7
	Yes	6	10.3
Employment status at the time of radiotherapy	Employed (incl. self‐employed)	48	82.8
	Unemployed	10	17.2
Employment status at the time of study participation	Employed (incl. self‐employed)	38	65.5
	Unemployed	20	34.5
Type of occupation before cancer treatment (*n* = 54)	Mainly intellectual work (“white collar”)	16	29.6
	Mainly physical work (“blue collar”)	18	33.3
	Equally intellectual and physical work	20	37.0
Returning to the same occupation as before cancer treatment (*n* = 38)	Yes	22	57.9
	No	16	42.1
Time until return to work following radiotherapy	< 3 months	9	15.5
	3–12 months	16	27.6
	> 12 months	12	20.7
	Unknown/no return to work	21	36.2
Professional return to work program	Yes	20	34.5
	No	20	34.5
	Unknown	18	31.0

	Median (IQR)
Weekly working time before cancer treatment (hours)	40.0 (25.0–40.0)
Weekly working time of adults who returned to work (hours)	38.8 (25.0–40.0)

Abbreviations: ECOG, Eastern Cooperative Oncology Group; IQR, interquartile range.

Before their cancer diagnosis, participants were almost equally distributed across occupational types, with approximately one third performing mainly intellectual work (16/54, 30%), one third mainly physical work (18/54, 33%), and one third engaging in mixed occupational tasks (20/54, 37%). At the time of the study, 38 patients (66%) were employed, with a median weekly working time of 38.8 h (IQR, 25–40). Among those who returned to work (*n* = 38), only 22 survivors (58%) were able to resume their previous occupation. A total of 20 participants (34%) reported access to a professional return to work program, while an equal proportion (34%) indicated that no such program was available, and for 18 respondents (31%) this information was unknown.

Nonresponders (*n* = 25) had the same median age (41 years) and a relatively comparable gender distribution (male: *n* = 18; 72%) as the study population. Consistent with study participants, the oral cavity (*n* = 7; 28%) and oropharynx (*n* = 5; 20%) were the two most common tumor localizations. However, the median time from the last radiotherapy fraction to questionnaire completion (or phone call for nonresponders) was higher in the nonresponder group (136 versus 82.5 months). The proportion of patients undergoing definitive radiotherapy (*n* = 12; 48%) was also substantially higher among nonresponders compared with study participants (*n* = 13; 22%).

### 3.2 Comparison of Quality of Life With the Age‐ and Gender‐Adjusted German Population

**FIGURE 1:**
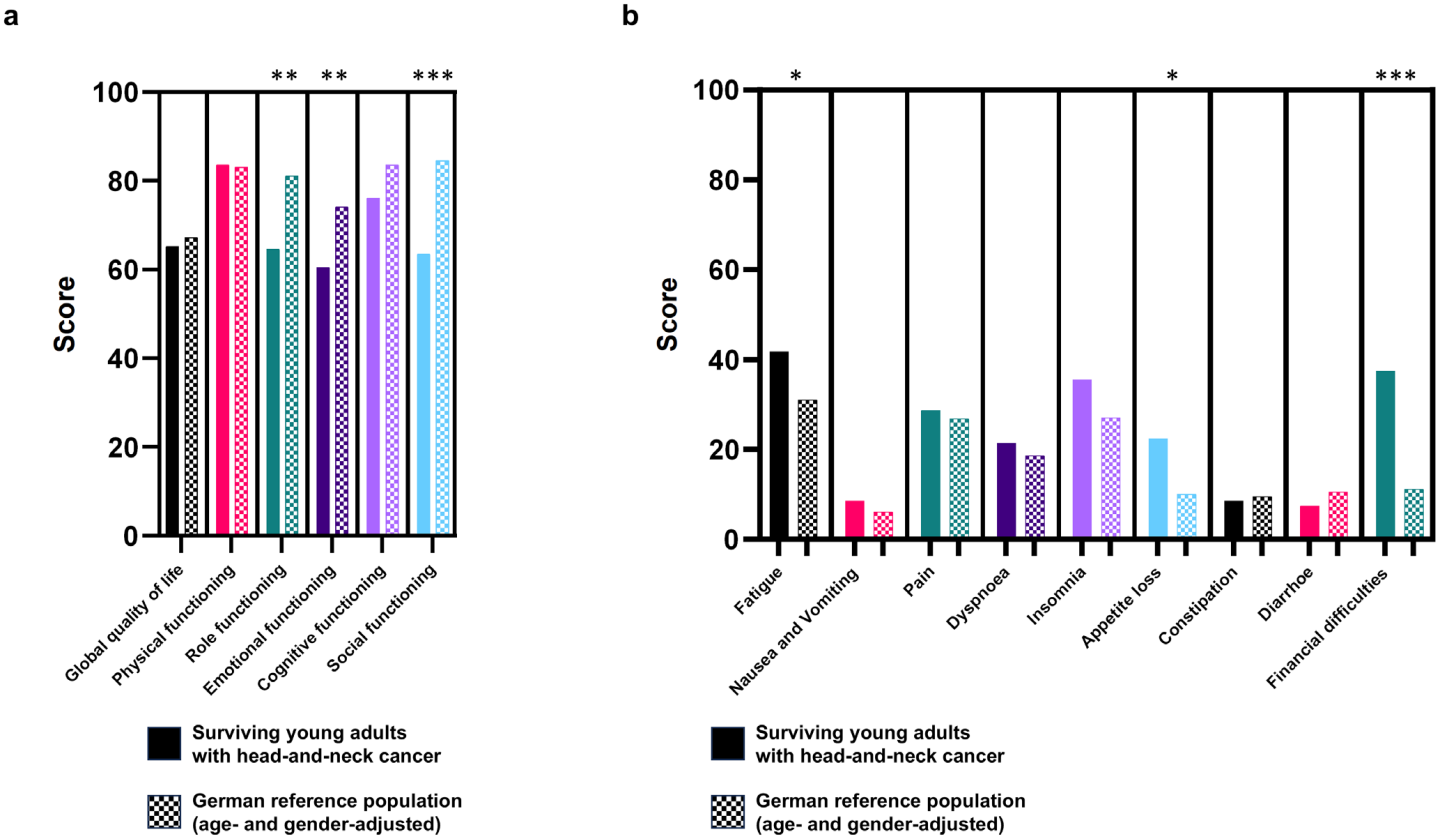
Quality of life comparison between surviving young adults with head‐and‐neck cancer and matched adults from the German general population (with the same age and sex). Groups were compared regarding the functioning subscales (a) and the symptom subscales/items (b) of the EORTC QLQ‐C30 questionnaire. Bars represent the mean values of the different subscales. General population norm data for Germany are obtained from Nolte et al. [18]. Unpaired two‐sided *t*‐tests were performed to compare both groups. *p < 0.05, **p < 0.01, ***p < 0.001.

To assess patient‐reported quality of life in the cohort of young adult head‐and‐neck cancer survivors, we used the EORTC QLQ‐C30 questionnaire. The mean global QoL score in our cohort was 65.0, comparable to the 67.2 points observed in the age‐ and gender‐adjusted German reference population (Figure 1). However, significant differences emerged between our cohort and the general population: Survivors reported statistically and clinically significantly lower scores in the domains of role functioning (64.6 vs. 81.1, *p* < 0.01), emotional functioning (60.5 vs. 74.1, *p* < 0.01), and social functioning (63.5 vs. 84.6, *p* < 0.001). Additionally, our cohort showed significantly higher scores on the symptom scales for fatigue (41.8 vs. 31.0, *p* < 0.05), appetite loss (22.4 vs. 10.0, *p* < 0.05), and financial difficulties (37.4 vs. 11.2, *p* < 0.001), indicating a greater symptom burden in these areas.

### 3.3 Association Between Return to Work and Quality of Life

**FIGURE 2:**
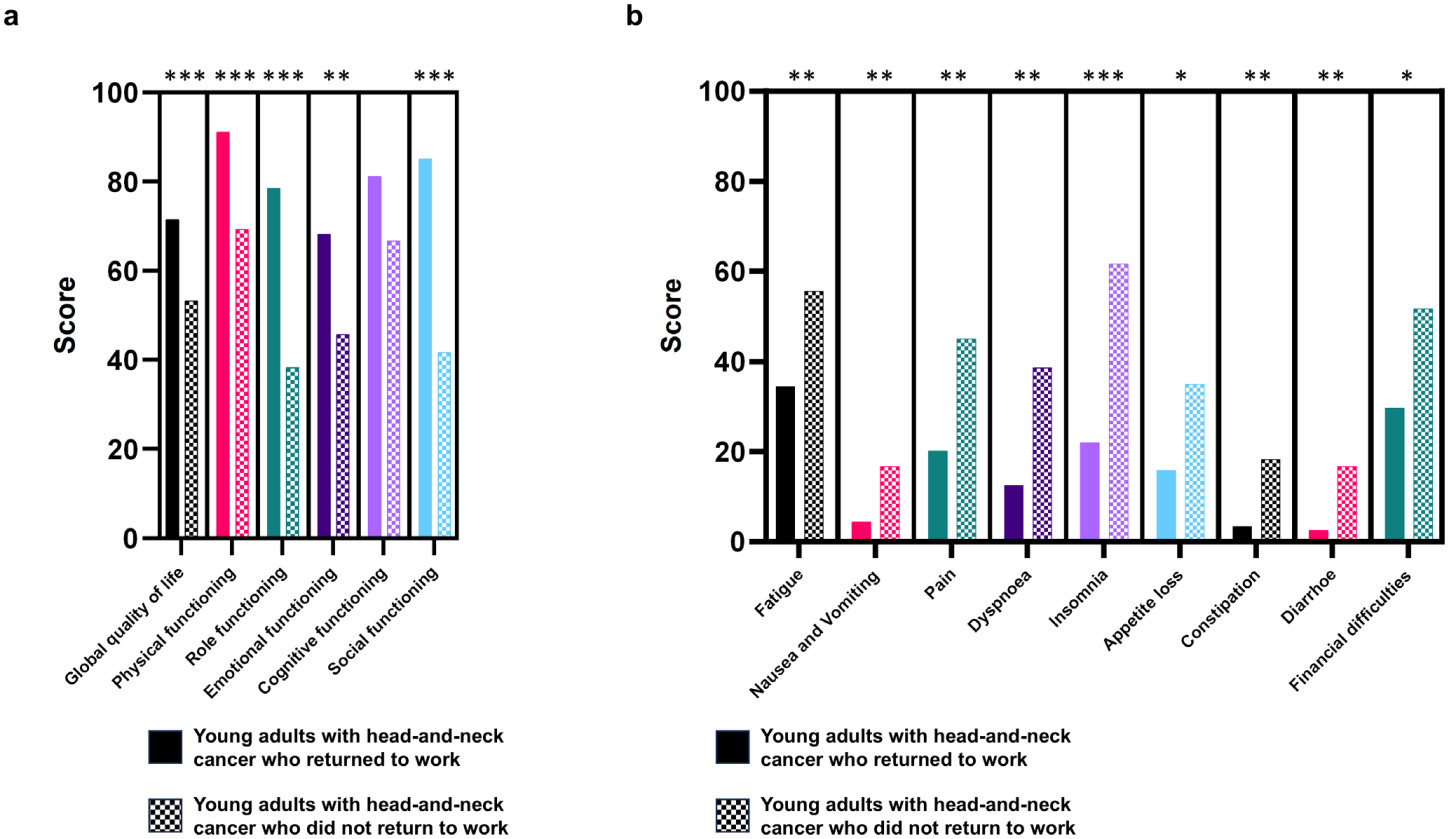
Quality of life comparison between surviving young adults with head‐and‐neck cancer who did return to work versus adults who did not return to work. Groups were compared regarding the functioning subscales (a) and the symptom subscales/items (b) of the EORTC QLQ‐C30 questionnaire. Bars represent the mean values of the different subscales. Mann–Whitney *U* tests were performed to compare both groups. *p < 0.05, **p < 0.01, ***p < 0.001.

We aimed to determine whether patient‐reported outcomes differed between those who returned to work after cancer treatment and those who did not. To investigate this, we compared the results of the EORTC QLQ‐C30 and the supplementary head‐and‐neck cancer module, EORTC QLQ‐HN43 (Figure 2). Patients who returned to work reported higher scores across all functioning scales except cognitive function and lower scores on all symptom scales of the EORTC QLQ‐C30. Survivors who did not return to work reported significantly worse outcomes across several EORTC QLQ‐HN43 subscales compared to those who resumed employment (Figure S2). The symptom scales for pain (*p* < 0.01), swallowing (*p* < 0.05), speech (*p* < 0.01), body image (*p* < 0.01), social eating (*p* < 0.05), sexuality (*p* < 0.05), skin problems (*p* < 0.01), coughing (*p* < 0.05), social contact (*p* < 0.001), swelling in the neck (*p* < 0.01), weight loss (*p* < 0.05), and wound healing (*p* < 0.001) were significantly more severe in the nonworking group.

Surviving young adults with head‐and‐neck cancer who returned to work also reported less depression, anxiety, and fear of cancer progression (Table 2). Adults without return to work exhibited higher levels of depression (PHQ‐9 mean = 11.6 vs. 5.1, *p* < 0.001) and anxiety (GAD‐7 mean = 8.7 vs. 4.2, *p* = 0.001), with a greater proportion experiencing moderate to severe symptoms in both domains. Fear of cancer progression (FoP‐Q‐SF mean = 34.3 vs. 28.6, *p* = 0.093) was also more pronounced in the group of patients who did not return to work, with 45% scoring ≥ 34 points compared to 32% in the return to work group. Work ability (WAI mean = 23.0 vs. 35.6, *p* < 0.001) was notably lower among those not returning to work. Distress, as measured by the NCCN Distress Thermometer, was significantly higher in surviving young head‐and‐neck cancer adults not returning to work (mean = 5.8 vs. 3.6, *p* = 0.015).

**TABLE 2:** Depression, anxiety, distress, fear of cancer progression, and work ability in surviving young adults with head‐and‐neck cancer undergoing radiotherapy.

Category	Subcategories	Entire population	Adults with RTW	Adults without RTW	*p*
PHQ‐9 (depression)	**Mean (SD)**	7.3 (5.6)	5.1 (3.5)	11.6 (6.4)	**< 0.001**
	**Median (IQR)**	6 (3–9.5)			
	Minimal (0–4)	20	18	2	
	Mild (5–9)	24	16	8	
	Moderate (10–14)	7	4	3	
	Moderately severe (15–19)	4	0	4	
	Severe (20–27)	3	0	3	
GAD‐7 (anxiety)	**Mean (SD)**	5.8 (4.7)	4.2 (3.6)	8.7 (5.3)	**0.001**
	**Median (IQR)**	4 (2.75–9)			
	Minimal (0–4)	30	25	5	
	Mild (5–9)	16	11	5	
	Moderate (10–14)	8	1	7	
	Severe (15–21)	4	1	3	
NCCN distress thermometer	**Mean (SD)**	4.4 (3.0)	3.6 (2.5)	5.8 (3.4)	**0.015**
	**Median (IQR)**	5 (2–7)			
	< 5 points	28	22	6	
	≥ 5 points	30	16	14	
FoP‐Q‐SF (fear of cancer progression)	**Mean (SD)**	30.6 (10.1)	28.6 (8.3)	34.3 (12.2)	0.093
	**Median (IQR)**	29 (21–37.25)			
	< 34 points	36	26	10	
	≥ 34 points	22	12	10	
WAI (work ability), *n* = 45	**Mean (SD)**	32.8 (8.4)	35.6 (6.2)	23.0 (7.9)	**< 0.001**
	**Median (IQR)**	33 (27–38)			
	Poor (7–27)	12	4	8	
	Moderate (28–36)	21	19	2	
	Good (37–43)	7	7	0	
	Excellent (44–49)	5	5	0	

*Note:* Mann–Whitney *U* tests were performed to compare both groups.

Abbreviations: IQR, interquartile range; RTW, return to work; SD, standard deviation. Bold *p* values represent significant findings (*p* < 0.05).

We analyzed correlations between work ability and several patient‐reported quality of life outcomes. Moderate negative correlations were found for distress (*r* = −0.48, *p* < 0.001), depression (*r* = −0.55, *p* < 0.001), and anxiety (*r* = −0.46, *p* < 0.001) (Table S2). There were no significant associations between work ability and global quality of life (*r* = 0.27, *p* = 0.079) or anxiety (*r* = −0.29, *p* = 0.079).

The number of patients who returned to work stratified by the previous type of occupation and the tumor site is shown in the Tables S3 and S4. These factors were not significantly associated with return to work, but results need to be interpreted cautiously due to the small number of subgroups. Numerically, a higher proportion of patients with primarily intellectual work returned to work (13/16, 81%) compared with those who initially performed mainly physical work (10/18, 56%). The multiple regression analysis, in which baseline patient characteristics and treatment parameters were entered, identified gender as a significant variable associated with return to work, with females showing higher odds of returning to work (β = −1.500, *p* < 0.05) (Table 3). Other variables, including age, usage of concurrent systemic treatment, type of radiotherapy (definitive or adjuvant), education level, partnership status, and ECOG performance status, were not significantly associated with return to work in our cohort.

**TABLE 3:** Variables associated with return to work after radiotherapy per regression analysis.

Variable	β	B	Lower 95% CI	Upper 95% CI	*p*
Age at the start of radiotherapy	−0.340	0.967	0.861	1.086	0.570
Gender (reference: female)	−1.500	0.223	0.052	0.959	**0.044**
Chemotherapy (reference: no)	0.582	1.790	0.438	7.309	0.417
Radiotherapy (reference: adjuvant)	0.183	1.201	0.219	6.596	0.883
Education (reference: no university degree)	1.077	2.935	0.274	31.411	0.373
Partnership at the time of treatment (reference: no)	−0.618	0.539	0.122	2.381	0.415
ECOG (reference: 0)	−1.052	0.349	0.091	1.332	0.304

*Note:* A complete‐case analysis (*n* = 56) with return to work as dependent variable was performed. The model exhibited a −2 Log‐Likelihood of 60.500, with a Cox & Snell *R* ^2^ of 0.182 and a Nagelkerke *R* ^2^ of 0.252. Bold *p* values represent significant findings (*p* < 0.05).

## 4 Discussion

In this study of young adult survivors of head‐and‐neck cancer that had been treated with radiotherapy, significant differences in patient‐reported outcomes were observed between those who returned to work and those who did not. Patients who were employed reported significantly better quality of life and lower levels of depression, anxiety, and fear of cancer progression compared to those without employment. Furthermore, fear of cancer progression and clinically relevant distress were prevalent in the entire cohort, with 38% of patients reporting a strong fear of cancer progression and 52% experiencing significant distress.

Interestingly, the global quality of life reported by the study cohort was comparable to the age‐ and gender‐adjusted general German population. However, response shift effects, selection bias, and survivorship bias may also have contributed to this finding [25]. Our results reveal clinically and statistically significant impairments in specific domains, including role, emotional, and social functioning. These findings align with prior research, indicating that head‐and‐neck cancer survivors often face long‐term challenges related to their interpersonal relationships, emotional resilience, and the ability to resume previous life roles [6, 26]. A previous study of 36 adolescent and young adult head‐and‐neck cancer survivors treated with radiotherapy in British Columbia between 1970 and 2010 reported significant late effects, including xerostomia (51%) and dysphagia (35%), as well as high levels of depression and anxiety, yet generally high health‐related quality of life—findings that are in line with our results [8].

The higher symptom burden in fatigue, appetite loss, and financial difficulties further highlights the multifaceted nature of survivorship challenges. Fatigue has been shown to negatively affect work ability in head‐and‐neck cancer survivors [27, 28]. Loss of appetite was another factor that was associated with employment after cancer treatment in head‐and‐neck cancer patients [29]. Financial difficulties, reported by over a third of participants, underscore the economic toll of cancer treatment and recovery. Previous studies have demonstrated that younger head‐and‐neck cancer patients often face more significant financial burdens than older adults [7, 30]. Psychological distress, anxiety, and depression were prevalent among the participants, with about half reporting significant distress. These findings are consistent with literature indicating that head‐and‐neck cancer survivors, irrespective of age, are at high risk of emotional and psychological difficulties [13]. The fear of cancer progression, experienced by nearly 40% of participants, reflects an additional source of ongoing psychological stress [31, 32]. This underscores the need for routine screening and interventions to address mental health issues in survivorship care, as these patients are a special subgroup among cancer survivors [33].

In our study, about two thirds of the study population returned to work after cancer treatment. This figure aligns with the findings of a meta‐analysis, which reported that the proportion of head‐and‐neck cancer survivors returning to work ranges from 32% to 90% [11]. Surviving head‐and‐neck cancer patients seem to have a higher risk of not returning to work than patients with other cancer types [34]. We observed that the employment status emerged as a critical factor influencing the overall well‐being of young adult survivors. Patients who returned to work reported better quality of life and reduced symptoms of depression, anxiety, and fear of cancer progression compared to their nonworking counterparts, consistent with previous studies [35, 36, 37]. Furthermore, depression, anxiety, fear of cancer progression as well as socially stigmatizing factors like speech or body image problems might severely interfere with the desire and ability to return to work [38]. Interestingly, gender was the only variable significantly associated with return to work in our regression analysis, with males showing lower odds of returning to work compared to females. A previous analysis of 398 head‐and‐neck cancer patients from Belgium also reported a significantly higher probability of women returning to work compared to men, at least in the univariate analysis [39]. Further research is needed to explore potential male‐specific barriers to workforce reintegration of young adults with head‐and‐neck cancer, such as societal roles, caregiving responsibilities, or differences in workplace discrimination. However, it is also important to consider differing risk profiles, as factors like alcohol and tobacco use—more prevalent among young male patients—may play a significant role regarding comorbidities and return to work [5].

### 4.1 Implications

The findings of this study underscore the need for tailored, multimodal survivorship programs for young adult head‐and‐neck cancer survivors. Given the high prevalence of distress, anxiety, depression, and fear of cancer progression observed in this population, integrating mental health services into routine survivorship care is essential. Hamilton et al. [40] evaluated the utility of survivorship care plans in adolescent and young adult head‐and‐neck cancer survivors and demonstrated that the majority of survivors and primary care providers found survivorship care plans helpful in understanding follow‐up needs and improving care transitions. Additionally, the significant impact of cancer and treatment‐related morbidity on work ability and return to work outcomes highlights the importance of careful initial treatment approaches (e.g., using state‐of‐the‐art radiotherapy techniques to reduce long‐term xerostomia and dysphagia) and of multiprofessional rehabilitation programs. These programs should emphasize skill‐building, workplace accommodation, and gradual reintegration to ensure long‐term employment success.

Addressing financial difficulties is another critical component of survivorship care [41, 42]. Financial counseling can assist survivors in navigating insurance claims, understanding employment rights, and accessing financial aid programs, thereby mitigating the economic burden of cancer and its treatment. Most head‐and‐neck cancer patients experience financial losses in Germany, despite the presence of a statutory health insurance system, due to out‐of‐pocket expenses and/or loss of income [42, 43]. In addition to out‐of‐pocket expenses, patients in Germany who are unable to return to work may apply for statutory disability insurance, which provides partial income replacement depending on the degree of work limitation. Access to these programs and the level of financial support may differ substantially from systems in other countries, which should be taken into account when interpreting our findings in an international context [44].

Furthermore, lifestyle and symptom management interventions are vital for improving physical functioning and addressing persistent symptoms such as fatigue, dry mouth, and sticky saliva, which can significantly affect quality of life and work ability [33]. Clarifying whether impaired quality of life prevents return to work, whether failure to return to work subsequently worsens quality of life, or whether both processes interact bidirectionally would help guide the development of targeted interventions. This would allow distinguishing whether improving posttreatment functioning and symptom burden should be prioritized, or whether strategies directly aimed at facilitating return to work may yield the greatest benefit [45]. A longitudinal study would therefore be required, ideally including repeated quality of life assessments early after radiotherapy to determine whether patients with more favorable recovery trajectories are more likely to return to work. Subsequently, the timing of return to work could be used to analyze longitudinal changes in quality of life, for example, whether returning to work stabilizes quality of life, further enhances it, or potentially worsens it if treatment‐related sequelae interfere with work performance.

### 4.2 Limitations

While providing a comprehensive analysis of quality of life and return to work based on a cross‐sectional observational study conducted at two comprehensive cancer centers, our study has several limitations that should be taken into account when interpreting the findings. First, as a cross‐sectional design was used, we were unable to assess changes in quality of life over time or make comparisons with the patients' quality of life prior to treatment. Second, selection bias cannot be excluded, as patients who chose to participate or returned the questionnaires may have had a better quality of life compared to those who declined to participate or were lost to follow‐up. Third, the self‐reported nature of the questionnaires may introduce response bias, as participants might have underreported or overreported their work ability. Fourth, subgroup analyses conducted among 58 surviving young head‐and‐neck cancer patients, while enabling the detection of significant differences between groups with fewer than 30 patients, increase the likelihood of random effects and elevate the probability of false positive reporting. Additionally, the effect estimates tend to be disproportionately high, as small sample sizes favor the overrepresentation of extreme distributions. Fifth, the sample size and the broad distribution of different head‐and‐neck cancer sites in our cohort did not allow robust analyses of site‐specific differences in return to work. Therefore, larger future studies will be required to evaluate potential variations in return‐to‐work outcomes by primary tumor site. Sixth, the inclusion of patients treated over such an extended time period introduces heterogeneity in treatment techniques and supportive care practices, which may confound some of the observed associations. Finally, since this was a cross‐sectional observational study conducted at two German comprehensive cancer centers, extrapolating our findings to other healthcare systems is challenging and should be done with caution.

## 5 Conclusions

The global quality of life of surviving young head‐and‐neck cancer patients after radiotherapy is comparable with the general age‐ and gender‐adjusted population. However, surviving young adults with head‐and‐neck cancer face significant challenges related to quality of life, psychological well‐being, and work reintegration. Our findings highlight the need for tailored survivorship programs that address these multifaceted issues. By incorporating psychological support, multiprofessional rehabilitation, financial counseling, and symptom management into routine care, healthcare providers may better support this vulnerable population with significant unmet needs in achieving long‐term recovery and well‐being. Future research in adequately powered, ideally international cohorts is warranted to identify the factors most strongly associated with symptom burden, quality of life, and workplace reintegration in young head‐and‐neck cancer patients after radiotherapy, and to clarify the directional relationships between these outcomes. Longitudinal and mixed‐methods studies are required to better analyze the relationship between return to work and quality of life.

## Author Contributions

**Charlotte Pannenbecker:** conceptualization (equal), formal analysis (lead), investigation (equal), methodology (equal), software (equal), validation (equal), visualization (equal), writing – original draft (supporting). **Clemens Seidel:** conceptualization (supporting), methodology (supporting), writing – review and editing (equal). **Jiadai Zou:** investigation (equal), writing – review and editing (equal). **Daniel Schnell:** writing – review and editing (equal). **Gunnar Wichmann:** writing – review and editing (lead). **Christoph Becker:** writing – review and editing (equal). **Sabine Klagges:** investigation (supporting), methodology (supporting). **Henning Schäfer:** writing – review and editing (equal). **Andreas Knopf:** writing – review and editing (equal). **Andreas Dietz:** writing – review and editing (equal). **Anca‐Ligia Grosu:** resources (equal), writing – review and editing (equal). **Anja Mehnert‐Theuerkauf:** methodology (equal), writing – review and editing (equal). **Nils H. Nicolay:** conceptualization (equal), methodology (equal), resources (equal), supervision (supporting), writing – original draft (supporting), writing – review and editing (equal). **Alexander Rühle:** conceptualization (lead), data curation (equal), formal analysis (equal), investigation (equal), methodology (equal), project administration (lead), resources (equal), software (equal), supervision (lead), validation (equal), visualization (equal), writing – original draft (lead).

## Funding

The study received partial funding from the Stiftung Roland‐Ernst‐Stiftung für Gesundheitswesen. The funding sources had no role in the design and conduct of the study or the decision to submit the manuscript for publication.

## Ethics Statement

The study was approved by the local institutional review boards in advance (reference numbers 077/23‐ek [Leipzig] and 371/20 with the amendment number 240159 [Freiburg]).

## Conflicts of Interest

The authors declare no conflicts of interest.

## Supporting information


**Figure S1:** Study flow diagram.
**Figure S2:** Quality of life comparison between surviving young adults with head‐and‐neck cancer who did return to work versus those who did not. Groups were compared regarding the symptom subscales/items of the EORTC QLQ‐HN43 questionnaire. Bars represent the mean values of the different subscales. Mann–Whitney *U* tests were performed to compare both groups. *p < 0.05, **p < 0.01, ***p < 0.001.
**Table S1:** Patient characteristics of nonresponders (*n* = 25). Patients either declined participation via telephone or did not send back the questionnaires. ECOG, Eastern Cooperative Oncology Group; IQR, interquartile range.
**Table S2:** Return to work stratified by type of previous work. Information on the type of previous work was available for 54 patients. Pearson's chi‐squared test did not show a significant association (*p* = 0.250).
**Table S3:** Return to work stratified by primary cancer site. Pearson's chi‐squared test did not show a significant association (*p* = 0.330).
**Table S4:** Correlation of Work Ability, assessed with the WAI questionnaire, with other patient‐reported outcome measures. Pearson correlation coefficient *r* with the according *p*‐value is indicated.

**Appendix S2:** STROBE statement—Checklist of items that should be included in reports of *cross‐sectional studies*.

## Data Availability

The data that support the findings of this study are available on request from the corresponding author. The dataset itself is not publicly available due to privacy or ethical restrictions.
