# Supplementary material for: Quality of Life and Return to Work After Radiotherapy in Young Adults With Head‐and‐Neck Cancer—A Bicentric Cross‐Sectional Observational Study
Source: Cancer Med. 2025 Dec 29;15(1):e71502. doi: 10.1002/cam4.71502 (PMC12745888; doi:10.1002/cam4.71502)
Supplement: Supplementary file 1 — Figure S1: Study flow diagram. Figure S2: Quality of life comparison between surviving young adults with head‐and‐neck cancer who did return to work versus those who did not. Groups were compared regarding the symptom subscales/items of the EORTC QLQ‐HN43 questionnaire. Bars represent the mean values of the different subscales. Mann–Whitney U tests were performed to compare both groups. *p < 0.05, **p < 0.01, ***p < 0.001. Table S1: Patient characteristics of nonresponders (n = 25). Patients either declined participation via telephone or did not send back the questionnaires. ECOG, Eastern Cooperative Oncology Group; IQR, interquartile range. Table S2: Return to work stratified by type of previous work. Information on the type of previous work was available for 54 patients. Pearson's chi‐squared test did not show a significant association (p = 0.250). Table S3: Return to work stratified by primary cancer site. Pearson's chi‐squared test did not show a significant association (p = 0.330). Table S4: Correlation of Work Ability, assessed with the WAI questionnaire, with other patient‐reported outcome measures. Pearson correlation coefficient r with the according p‐value is indicated. [file CAM4-15-e71502-s001.docx]

**Supplementary Data for the Manuscript**

**Quality of life and return to work after radiotherapy in young adults with head-and-neck cancer – A bicentric cross-sectional observational study**

**Return to work questionnaire (English and German version).**

English translation:

1. Please state your highest educational qualification here:

- Secondary school
- High school diploma
- University degree
- Others

Occupation **before the cancer disease**:

1. Were you employed at the time of the cancer diagnosis?

- Yes
- No

1. Were you living in a partnership at the time of the cancer diagnosis?

- Yes
- No

1. Were you at work

- Mainly intellectually active?
- Mainly physically active?
- Were you equally intellectually and physically active?

1. Did you work as a freelancer or as an employee?

- Freelance/self-employed
- Salaried employment
- Other (training, studies)

6. Please state the average number of weekly work hours before the cancer disease:

Occupation **after the cancer disease**:

7. Did you undergo a professional return to work program?

- Yes
- No

8. Are you currently living in a partnership?

- Yes
- No

9. Are you currently employed?

- Yes
- No

The following questions relate to patients **who have already been able to return to work**:

10. Are you fulfilling the same activity as before the illness?

- Yes
- No

11. When did you return to work?

- Within the first 3 months after completing treatment
- Within 3 - 12 months after completing treatment
- Later than 12 months after completing therapy

12. Do you currently work as a freelancer or as an employee?

- Freelance/self-employed
- Salaried employment
- Other (training, studies)

13. Please state the current average number of weekly work hours:

German original version:

1. Bitte geben Sie hier Ihren höchsten Bildungsabschluss an:
   - Volks-/Hauptschulabschluss, Mittlere Reife
   - (Fach)Abitur
   - Universitätsabschluss
   - Sonstiges

Berufstätigkeit **vor der Tumorerkrankung**:

1. Waren Sie zum Zeitpunkt der Tumordiagnose berufstätig?
   - Ja
   - Nein
2. Lebten Sie zum Zeitpunkt der Tumordiagnose in einer Partnerschaft
   - Ja
   - Nein
3. Waren Sie bei Ihrer Arbeit
   - Vorwiegend geistig tätig?
   - Vorwiegend körperlich tätig?
   - Gleichermaßen geistig und körperlich tätig?
4. Haben Sie freiberuflich oder angestellt gearbeitet?
   - Freiberuflich/Selbstständig
   - Angestelltes Arbeitsverhältnis
   - Sonstiges (Ausbildung, Studium)
5. Bitte geben Sie die Anzahl der durchschnittlichen wöchentlichen Arbeitsstunden an:

Berufstätigkeit **nach der Tumorerkrankung**:

1. Haben Sie eine berufliche Wiedereingliederungsmaßnahme erhalten?
   - Ja
   - Nein
2. Leben Sie momentan in einer Partnerschaft?
   - Ja
   - Nein
3. Sind Sie zum aktuellen Zeitpunkt berufstätig?
   - Ja
   - Nein

Die folgenden Fragen beziehen sich auf Patientinnen und Patienten, die ihre **Berufstätigkeit bereits wieder aufnehmen konnten**:

1. Üben Sie dieselbe Tätigkeit aus wie vor der Erkrankung?
   - Ja
   - Nein
2. Wann haben Sie Ihre Berufstätigkeit wieder aufgenommen
   - Innerhalb der ersten 3 Monate nach Abschluss der Therapie
   - Innerhalb von 3 – 12 Monaten nach Abschluss der Therapie
   - Später als 12 Monate nach Abschluss der Therapie
3. Arbeiten Sie freiberuflich oder angestellt?
   - Freiberuflich/Selbstständig
   - Angestelltes Arbeitsverhältnis
   - Sonstiges (Ausbildung, Studium)

13. Bitte geben Sie die Anzahl der durchschnittlichen wöchentlichen Arbeitsstunden an:

**Supplementary Figure 1. Study flow diagram.**


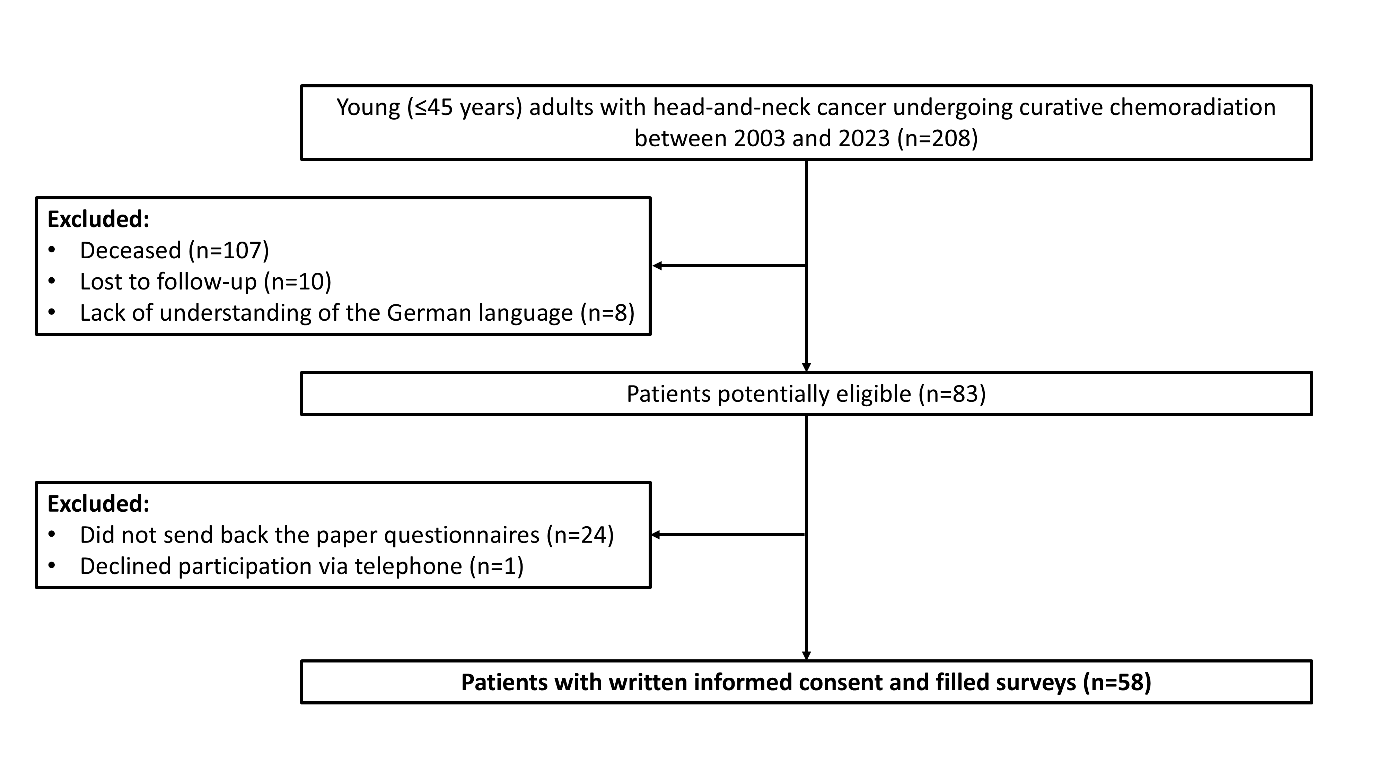


**Supplementary Figure 2.** **Quality of life comparison between surviving young adults with head-and-neck cancer who did return to work versus those who did not.** Groups were compared regarding the symptom subscales/items of the EORTC QLQ-HN43 questionnaire. Bars represent the mean values of the different subscales. Mann-Whitney U tests were performed to compare both groups. **p*<0.05, ***p*<0.01, ****p*<0.001.


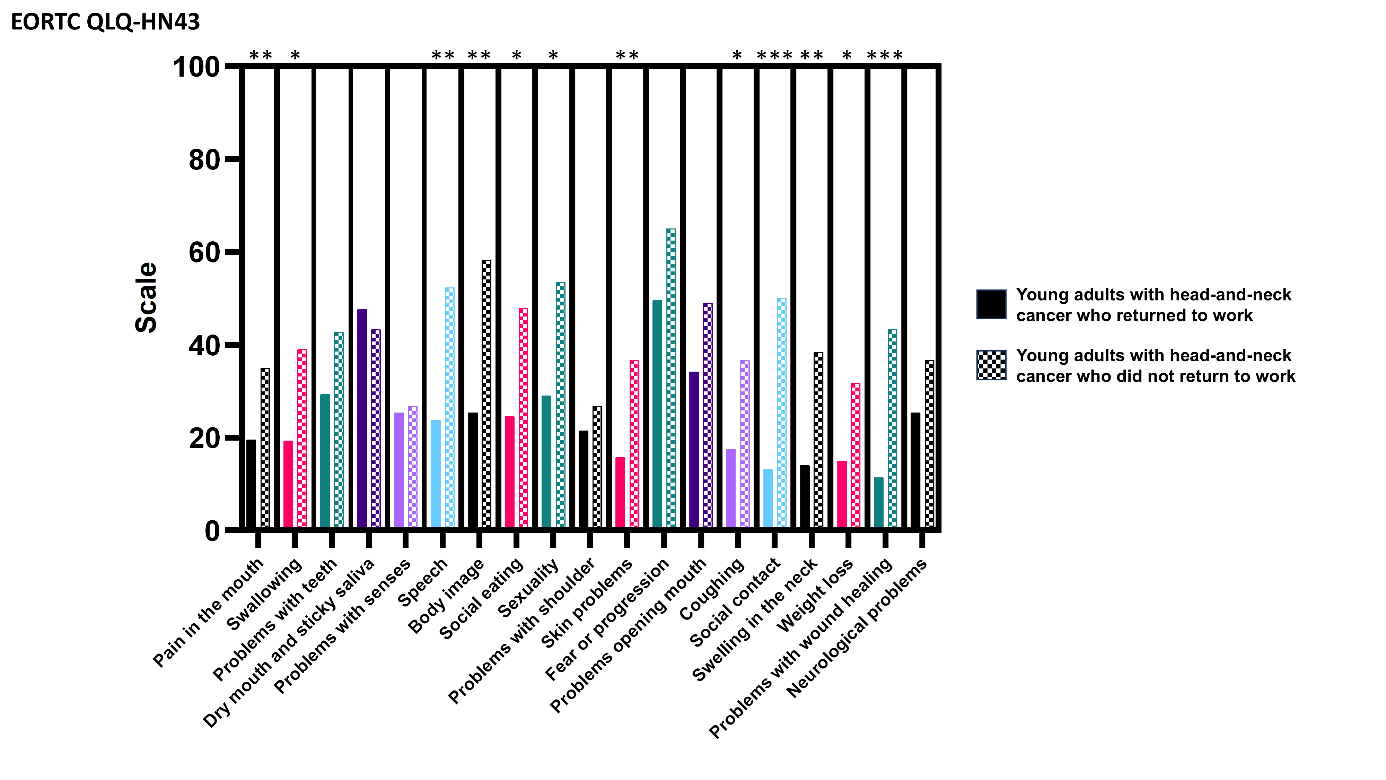


**Supplementary Table 1. Patient characteristics of non-responders (n=25).** Patients either declined participation via telephone or did not send back the questionnaires. ECOG, Eastern Cooperative Oncology Group; IQR, interquartile range.

|  | | Median IQR) | |
| --- | --- | --- | --- |
| Age at start of first radiotherapy course [years] | | 41 (39-43.5) | |
| Radiotherapy treatment fractions | | 32 (30-34.5) | |
| Median time between last fraction of radiotherapy and telephone call regarding study participation [months] | | 136 (74-156) | |
|  | | **n** | **%** |
| Gender | Male | 18 | 72 |
|  | Female | 7 | 28 |
| Health insurance | Public health insurance | 19 | 76 |
|  | Private health insurance | 6 | 24 |
| Smoking status at the time of radiotherapy | Never smoker | 6 | 24 |
|  | Former/current smoker | 15 | 60 |
|  | Unknown | 4 | 16 |
| Performance status at the beginning of radiotherapy | ECOG 0 | 14 | 56 |
|  | ECOG 1 | 11 | 44 |
| Primary cancer site | Oral cavity | 7 | 28 |
|  | Oropharynx | 5 | 20 |
|  | Hypopharynx | 1 | 4 |
|  | Larynx | 5 | 20 |
|  | Nasopharynx | 0 | 0 |
|  | Salivary gland | 2 | 8 |
|  | Nasal cavity/paranasal sinus | 2 | 8 |
|  | Cancer in the neck with an unknown primary | 1 | 4 |
|  | Multilevel pharynx | 2 | 8 |
| Type of radiotherapy | Definitive | 12 | 48 |
|  | Adjuvant | 13 | 52 |
| Concurrent systemic treatment | Concomitant systemic treatment | 14 | 56 |
|  | No concomictant systemic treatment | 11 | 44 |
| Radiotherapy-induced chronic grade 3/4 toxicity | No | 16 | 64 |
|  | Yes | 4 | 16 |
|  | Unknown | 5 | 20 |
| Cancer recurrence | No | 20 | 80 |
|  | Yes | 4 | 16 |
|  | Unknown | 1 | 4 |

**Supplementary Table 2. Return to work stratified by type of previous work.** Information on the type of previous work was available for 54 patients. Pearson's chi-squared test did not show a significant association (*p*=0.250).

| **Type of previous work** | **Return to work** | |
| --- | --- | --- |
|  | **Yes** | **No** |
| Mainly intellectual work (“white collar”) | 13 | 3 |
| Mainly physical work (“blue collar”) | 10 | 8 |
| Equally intellectual and physical work | 12 | 8 |

**Supplementary Table 3. Return to work stratified by primary cancer site.** Pearson's chi-squared test did not show a significant association (*p*=0.330).

| **Primary cancer site** | **Return to work** | |
| --- | --- | --- |
|  | **Yes** | **No** |
| Oral cavity | 12 | 9 |
| Oropharynx | 9 | 3 |
| Hypopharynx | 0 | 2 |
| Larynx | 1 | 2 |
| Nasopharynx | 4 | 1 |
| Salivary gland | 5 | 2 |
| Nasal cavity/paranasal sinus | 3 | 1 |
| Head-and-neck cancer of unknown primary | 3 | 0 |
| Multilevel pharynx | 1 | 0 |

**Supplementary Table 4. Correlation of Work Ability, assessed with the WAI questionnaire, with other patient-reported outcome measures.** Pearson correlation coefficient *r* with the according *p*-value is indicated.

| **Variable** | ***r*** | ***p*** |
| --- | --- | --- |
| Global quality of life | 0.27 | 0.079 |
| Distress | - 0.48 | **<0.001** |
| Depression | - 0.55 | **<0.001** |
| Anxiety | - 0.46 | **<0.001** |
| Fear of cancer recurrence | - 0.29 | 0.079 |
